# Poly[bis­(μ_4_-acetato-κ^4^ *O*:*O*:*O*′:*O*′)bis­(μ_3_-acetato-κ^3^ *O*:*O*:*O*)(μ_2_-acetato-κ^2^ *O*:*O*′)(μ_2_-acetic acid-κ^2^ *O*:*O*′)di-μ-aqua­copper(II)tris­odium]

**DOI:** 10.1107/S1600536810035683

**Published:** 2010-09-11

**Authors:** Xiang-Yu Jiang, Xi-Gui Yue

**Affiliations:** a Alan G. MacDiarmid Institute, Jilin University, Changchun 130012, People’s Republic of China

## Abstract

In the title compound, [CuNa_3_(CH_3_CO_2_)_5_(CH_3_COOH)(H_2_O)_2_]_*n*_, the Cu^II^ atom lies on an inversion center and is coordinated by four O atoms from four acetate ligands, leading to a square-planar geometry. One Na^I^ atom, lying on an inversion center, is coordinated by four O atoms from four acetate ligands and two bridging water mol­ecules in a distorted octa­hedral geometry. The other Na^I^ atom is coordinated by five O atoms from five acetate ligands and a bridging water mol­ecule. A hy­droxy H atom lies on a twofold rotation axis and is shared by two acetate ligands. The crystal packing exhibits a polymeric layer parallel to (100), which is further stablized by intra­layer O—H⋯O hydrogen bonds. The layers are linked by inter­layer O—H⋯O hydrogen bonds.

## Related literature

For related structures, see: Chiari *et al.* (1988 ▶); Vives *et al.* (2003 ▶).
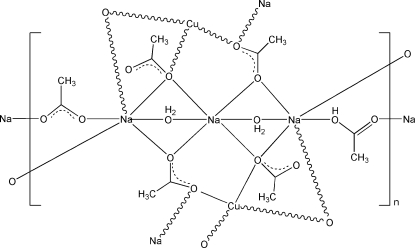


## Experimental

### 

#### Crystal data

[CuNa_3_(C_2_H_3_O_2_)_5_(C_2_H_4_O_2_)(H_2_O)_2_]
*M* *_r_* = 523.81
Monoclinic, 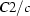

*a* = 14.571 (6) Å
*b* = 6.768 (3) Å
*c* = 22.653 (10) Å
β = 103.426 (19)°
*V* = 2173.0 (16) Å^3^
*Z* = 4
Mo *K*α radiation
μ = 1.13 mm^−1^
*T* = 290 K
0.27 × 0.25 × 0.23 mm

#### Data collection

Rigaku R-AXIS RAPID diffractometer
Absorption correction: multi-scan (*ABSCOR*; Higashi, 1995 ▶) *T*_min_ = 0.752, *T*_max_ = 0.777
10214 measured reflections
2493 independent reflections
2192 reflections with *I* > 2σ(*I*)
*R*_int_ = 0.023

#### Refinement

*R*[*F* ^2^ > 2σ(*F* ^2^)] = 0.023
*wR*(*F* ^2^) = 0.068
*S* = 1.03
2493 reflections
142 parameters
H-atom parameters constrained
Δρ_max_ = 0.30 e Å^−3^
Δρ_min_ = −0.32 e Å^−3^

### 

Data collection: *RAPID-AUTO* (Rigaku, 1998 ▶); cell refinement: *RAPID-AUTO*; data reduction: *CrystalStructure* (Rigaku/MSC, 2002 ▶); program(s) used to solve structure: *SHELXS97* (Sheldrick, 2008 ▶); program(s) used to refine structure: *SHELXL97* (Sheldrick, 2008 ▶); molecular graphics: *PLATON* (Spek, 2009 ▶) and *DIAMOND* (Brandenburg, 1999 ▶); software used to prepare material for publication: *SHELXL97*.

## Supplementary Material

Crystal structure: contains datablocks global, I. DOI: 10.1107/S1600536810035683/hy2345sup1.cif

Structure factors: contains datablocks I. DOI: 10.1107/S1600536810035683/hy2345Isup2.hkl

Additional supplementary materials:  crystallographic information; 3D view; checkCIF report

## Figures and Tables

**Table 1:** Hydrogen-bond geometry (Å, °)

*D*—H⋯*A*	*D*—H	H⋯*A*	*D*⋯*A*	*D*—H⋯*A*
O7—H7*A*⋯O1	0.79	2.05	2.835 (2)	172
O7—H7*B*⋯O1^i^	0.80	2.03	2.8104 (19)	168

Symmetry code: (i) 
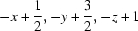
.

**Fig. 1.**
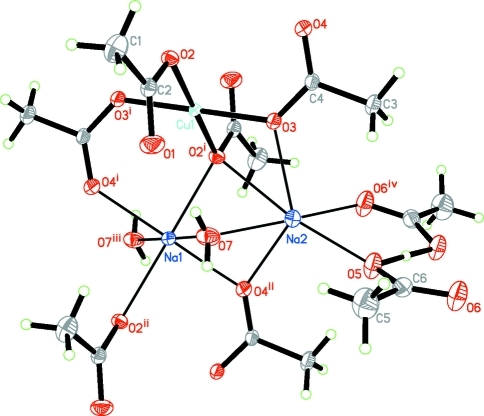
The structure of the title compound. Displacement ellipsoids are drawn at the 30% probability level. [Symmetry codes: (i) -x+1, -y+2, -z+1; (ii) x, y-1, z; (iii) -x+1, -y+1, -z+1; (iv) -x+1, y, -z+1/2.]

**Fig. 2.**
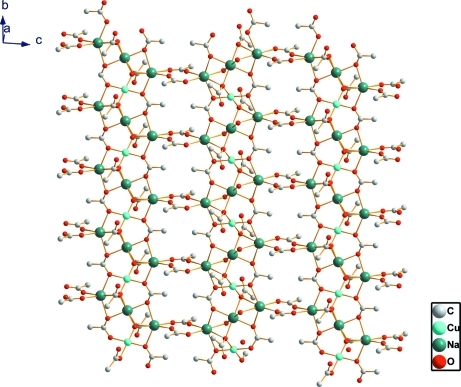
A view of the two-dimensional structure in the title compound.

## supplementary crystallographic information

### Comment

There has been increasing interest in the study of copper-containing complexes due to their various coordination styles and potential applications. We report here the crystal structure of the title compound, a new copper complex with acetate.

In the title compound, as shown in Fig. 1, the Cu^II^ atom lies on an inversion center and is four-coordinated by four O atoms from four acetate ligands, forming a square-planar coordination geometry. In a comparison with the tiltle compound, the complexes previously reported (Chiari *et al.*, 1988; Vives *et al.*, 2003) show different coordination behaviors of the central Cu^II^ ion. The two Na^I^ ions are each coordinated by six O atoms, forming a distorted octahedral coordination geometry. Na1 atom lies on an inversion center, while Na2 atom is on a general position. In the crystal structure, the metal ions, acetate ligands and water molecules are connected each other, forming a two-dimensional network (Fig. 2). The crystal packing is further stabilized by intermolecular O—H···O hydrogen bonds (Table 1).

### Experimental

The title compound was prepared as follows: copper acetate dihydrate (2.18 g, 0.01 mol) was added to a solution of glacial acetic acid (3 ml) in 15 ml water. Then 10 ml NaOH (3 mol/*L*) was added into the mixture. The mixture was heated and stirred for half an hour and then filtered. The filtrate was allowed to stand at room temperature for several days, giving blue block-shaped crystals.

### Refinement

C-bound H atoms were placed in calculated positions and refined as riding atoms, with C—H = 0.96 Å and with *U*_iso_(H) = 1.5*U*_eq_(C). H atoms of water and carboxyl group are located in a difference Fourier map and refined as riding, with *U*_iso_(H) = 1.5*U*_eq_(O).

### Crystal data

**Table:**

[CuNa_3_(C_2_H_3_O_2_)_5_(C_2_H_4_O_2_)(H_2_O)_2_]	*F*(000) = 1076
*M**_r_* = 523.81	*D*_x_ = 1.601 Mg m^−^^3^
Monoclinic, *C*2/*c*	Mo *K*α radiation, λ = 0.71073 Å
Hall symbol: -C 2yc	Cell parameters from 8924 reflections
*a* = 14.571 (6) Å	θ = 3.0–27.5°
*b* = 6.768 (3) Å	µ = 1.13 mm^−^^1^
*c* = 22.653 (10) Å	*T* = 290 K
β = 103.426 (19)°	Block, blue
*V* = 2173.0 (16) Å^3^	0.27 × 0.25 × 0.23 mm
*Z* = 4	

### Data collection

**Table:**

Rigaku R-AXIS RAPID diffractometer	2493 independent reflections
Radiation source: rotaton anode	2192 reflections with *I* > 2σ(*I*)
graphite	*R*_int_ = 0.023
ω scans	θ_max_ = 27.5°, θ_min_ = 3.0°
Absorption correction: multi-scan (*ABSCOR*; Higashi, 1995)	*h* = −18→18
*T*_min_ = 0.752, *T*_max_ = 0.777	*k* = −8→8
10214 measured reflections	*l* = −29→29

### Refinement

**Table:**

Refinement on *F*^2^	Primary atom site location: structure-invariant direct methods
Least-squares matrix: full	Secondary atom site location: difference Fourier map
*R*[*F*^2^ > 2σ(*F*^2^)] = 0.023	Hydrogen site location: inferred from neighbouring sites
*wR*(*F*^2^) = 0.068	H-atom parameters constrained
*S* = 1.03	*w* = 1/[σ^2^(*F*_o_^2^) + (0.039*P*)^2^ + 1.7139*P*] where *P* = (*F*_o_^2^ + 2*F*_c_^2^)/3
2493 reflections	(Δ/σ)_max_ = 0.011
142 parameters	Δρ_max_ = 0.30 e Å^−^^3^
0 restraints	Δρ_min_ = −0.32 e Å^−^^3^

### Special details

**Table:**

Experimental. (See detailed section in the paper)

### Fractional atomic coordinates and isotropic or equivalent isotropic displacement parameters (Å^2^)

**Table:**

	*x*	*y*	*z*	*U*_iso_*/*U*_eq_	
C1	0.26112 (13)	1.3050 (3)	0.41200 (9)	0.0451 (5)	
H1A	0.2567	1.2814	0.3696	0.068*	
H1B	0.2834	1.4370	0.4221	0.068*	
H1C	0.2000	1.2893	0.4206	0.068*	
C2	0.32852 (10)	1.1606 (2)	0.44894 (7)	0.0252 (3)	
C3	0.49416 (15)	1.1930 (3)	0.67768 (7)	0.0400 (4)	
H3A	0.4943	1.3232	0.6945	0.060*	
H3B	0.5462	1.1188	0.7010	0.060*	
H3C	0.4361	1.1277	0.6788	0.060*	
C4	0.50335 (11)	1.2077 (2)	0.61301 (6)	0.0237 (3)	
C5	0.25974 (15)	0.7130 (4)	0.69424 (11)	0.0614 (6)	
H5A	0.2259	0.5966	0.7010	0.092*	
H5B	0.2658	0.7151	0.6529	0.092*	
H5C	0.2259	0.8282	0.7020	0.092*	
C6	0.35605 (13)	0.7116 (3)	0.73620 (8)	0.0352 (4)	
Cu1	0.5000	1.0000	0.5000	0.01852 (8)	
Na1	0.5000	0.5000	0.5000	0.02776 (19)	
Na2	0.50180 (5)	0.70788 (10)	0.62598 (3)	0.03245 (16)	
O1	0.30176 (9)	0.99791 (19)	0.46272 (7)	0.0430 (3)	
O2	0.41529 (7)	1.21412 (16)	0.46414 (5)	0.0244 (2)	
O3	0.49001 (8)	1.04618 (17)	0.58292 (5)	0.0282 (2)	
O4	0.52322 (10)	1.36734 (18)	0.59343 (5)	0.0374 (3)	
O5	0.42574 (9)	0.6909 (2)	0.71094 (6)	0.0455 (3)	
H5	0.5000	0.6936	0.7500	0.068*	
O6	0.36605 (10)	0.7272 (3)	0.79096 (6)	0.0517 (4)	
O7	0.37443 (8)	0.67127 (19)	0.53741 (6)	0.0382 (3)	
H7B	0.3291	0.6111	0.5410	0.057*	
H7A	0.3518	0.7657	0.5191	0.057*	

### Atomic displacement parameters (Å^2^)

**Table:**

	*U*^11^	*U*^22^	*U*^33^	*U*^12^	*U*^13^	*U*^23^
C1	0.0326 (9)	0.0591 (13)	0.0409 (10)	0.0154 (9)	0.0033 (7)	0.0106 (9)
C2	0.0234 (7)	0.0315 (8)	0.0213 (7)	0.0002 (6)	0.0061 (5)	0.0000 (6)
C3	0.0630 (12)	0.0376 (10)	0.0218 (8)	−0.0002 (9)	0.0148 (8)	0.0001 (7)
C4	0.0287 (7)	0.0242 (7)	0.0184 (6)	0.0026 (6)	0.0058 (5)	0.0003 (6)
C5	0.0415 (11)	0.0837 (18)	0.0532 (13)	0.0000 (11)	−0.0011 (10)	−0.0001 (12)
C6	0.0386 (9)	0.0332 (9)	0.0328 (8)	−0.0008 (7)	0.0063 (7)	0.0024 (7)
Cu1	0.02176 (13)	0.01720 (13)	0.01633 (12)	−0.00190 (9)	0.00390 (9)	0.00037 (9)
Na1	0.0418 (5)	0.0207 (4)	0.0213 (4)	−0.0071 (4)	0.0084 (4)	−0.0001 (3)
Na2	0.0433 (4)	0.0303 (3)	0.0235 (3)	−0.0038 (3)	0.0071 (3)	−0.0006 (3)
O1	0.0336 (6)	0.0445 (8)	0.0488 (8)	−0.0151 (5)	0.0055 (6)	0.0105 (6)
O2	0.0228 (5)	0.0218 (5)	0.0271 (5)	−0.0013 (4)	0.0026 (4)	0.0006 (4)
O3	0.0426 (6)	0.0230 (5)	0.0205 (5)	−0.0008 (5)	0.0102 (4)	−0.0001 (4)
O4	0.0617 (8)	0.0254 (6)	0.0243 (5)	−0.0066 (6)	0.0084 (5)	0.0017 (5)
O5	0.0401 (7)	0.0691 (10)	0.0281 (6)	−0.0009 (7)	0.0095 (5)	−0.0013 (6)
O6	0.0430 (7)	0.0822 (12)	0.0316 (7)	0.0048 (7)	0.0122 (6)	0.0004 (7)
O7	0.0270 (6)	0.0361 (7)	0.0502 (7)	−0.0062 (5)	0.0060 (5)	0.0030 (6)

### Geometric parameters (Å, °)

**Table:**

C1—C2	1.496 (2)	Cu1—O3	1.9426 (13)
C1—H1A	0.9600	Cu1—O2	1.9540 (12)
C1—H1B	0.9600	Cu1—Na1^i^	3.3842 (14)
C1—H1C	0.9600	Cu1—Na2^ii^	3.4669 (13)
C2—O1	1.232 (2)	Na1—O4^iii^	2.2512 (14)
C2—O2	1.2831 (18)	Na1—O2^iii^	2.3371 (13)
C3—C4	1.505 (2)	Na1—O7	2.4759 (15)
C3—H3A	0.9600	Na1—Na2^iv^	3.1765 (13)
C3—H3B	0.9600	Na2—O6^v^	2.3627 (18)
C3—H3C	0.9600	Na2—O7	2.4088 (16)
C4—O4	1.228 (2)	Na2—O5	2.4362 (17)
C4—O3	1.2792 (19)	Na2—O4^iii^	2.4618 (17)
C5—C6	1.501 (3)	Na2—O3	2.4792 (16)
C5—H5A	0.9600	Na2—O2^ii^	2.6554 (15)
C5—H5B	0.9600	O5—H5	1.2286
C5—H5C	0.9600	O7—H7B	0.7965
C6—O6	1.220 (2)	O7—H7A	0.7910
C6—O5	1.284 (2)		
			
C2—C1—H1A	109.5	O2^ii^—Na1—O7	82.30 (5)
C2—C1—H1B	109.5	O2^iii^—Na1—O7	97.70 (5)
H1A—C1—H1B	109.5	O7^iv^—Na1—O7	180.0
C2—C1—H1C	109.5	O4^ii^—Na1—Na2^iv^	50.50 (4)
H1A—C1—H1C	109.5	O4^iii^—Na1—Na2^iv^	129.50 (4)
H1B—C1—H1C	109.5	O2^ii^—Na1—Na2^iv^	124.93 (3)
O1—C2—O2	122.32 (14)	O2^iii^—Na1—Na2^iv^	55.07 (3)
O1—C2—C1	121.35 (15)	O7^iv^—Na1—Na2^iv^	48.52 (3)
O2—C2—C1	116.31 (15)	O7—Na1—Na2^iv^	131.48 (4)
C4—C3—H3A	109.5	O4^ii^—Na1—Cu1^iii^	113.51 (3)
C4—C3—H3B	109.5	O4^iii^—Na1—Cu1^iii^	66.49 (3)
H3A—C3—H3B	109.5	O2^ii^—Na1—Cu1^iii^	145.89 (3)
C4—C3—H3C	109.5	O2^iii^—Na1—Cu1^iii^	34.11 (3)
H3A—C3—H3C	109.5	O7^iv^—Na1—Cu1^iii^	62.08 (3)
H3B—C3—H3C	109.5	O7—Na1—Cu1^iii^	117.92 (3)
O4—C4—O3	125.37 (14)	Na2^iv^—Na1—Cu1^iii^	63.708 (19)
O4—C4—C3	119.44 (15)	O6^v^—Na2—O7	175.43 (6)
O3—C4—C3	115.18 (14)	O6^v^—Na2—O5	79.03 (6)
C6—C5—H5A	109.5	O7—Na2—O5	104.44 (6)
C6—C5—H5B	109.5	O6^v^—Na2—O4^iii^	98.84 (6)
H5A—C5—H5B	109.5	O7—Na2—O4^iii^	77.35 (5)
C6—C5—H5C	109.5	O5—Na2—O4^iii^	107.75 (5)
H5A—C5—H5C	109.5	O6^v^—Na2—O3	103.39 (6)
H5B—C5—H5C	109.5	O7—Na2—O3	78.33 (5)
O6—C6—O5	122.86 (17)	O5—Na2—O3	110.60 (5)
O6—C6—C5	121.13 (18)	O4^iii^—Na2—O3	138.49 (5)
O5—C6—C5	116.00 (17)	O6^v^—Na2—O2^ii^	99.73 (6)
O3^ii^—Cu1—O3	180.0	O7—Na2—O2^ii^	77.30 (5)
O3^ii^—Cu1—O2^ii^	95.78 (5)	O5—Na2—O2^ii^	171.22 (5)
O3—Cu1—O2^ii^	84.22 (5)	O4^iii^—Na2—O2^ii^	81.02 (5)
O3^ii^—Cu1—O2	84.22 (5)	O3—Na2—O2^ii^	61.06 (4)
O3—Cu1—O2	95.78 (5)	C2—O2—Cu1	112.95 (10)
O2^ii^—Cu1—O2	180.00 (6)	C2—O2—Na1^i^	137.32 (10)
O3^ii^—Cu1—Na1^i^	99.26 (3)	Cu1—O2—Na1^i^	103.76 (5)
O3—Cu1—Na1^i^	80.74 (3)	C2—O2—Na2^ii^	116.47 (9)
O2^ii^—Cu1—Na1^i^	137.87 (3)	Cu1—O2—Na2^ii^	96.36 (5)
O2—Cu1—Na1^i^	42.13 (3)	Na1^i^—O2—Na2^ii^	78.74 (4)
O3^ii^—Cu1—Na2^ii^	44.26 (4)	C4—O3—Cu1	128.07 (10)
O3—Cu1—Na2^ii^	135.74 (4)	C4—O3—Na2	126.27 (10)
O2^ii^—Cu1—Na2^ii^	130.43 (4)	Cu1—O3—Na2	102.59 (5)
O2—Cu1—Na2^ii^	49.57 (4)	C4—O4—Na1^i^	133.95 (10)
Na1^i^—Cu1—Na2^ii^	55.23 (2)	C4—O4—Na2^i^	131.20 (11)
O4^ii^—Na1—O4^iii^	180.0	Na1^i^—O4—Na2^i^	84.62 (5)
O4^ii^—Na1—O2^ii^	87.05 (5)	C6—O5—Na2	154.01 (12)
O4^iii^—Na1—O2^ii^	92.95 (5)	C6—O5—H5	109.4
O4^ii^—Na1—O2^iii^	92.95 (5)	Na2—O5—H5	94.7
O4^iii^—Na1—O2^iii^	87.05 (5)	C6—O6—Na2^v^	133.48 (13)
O2^ii^—Na1—O2^iii^	180.0	Na2—O7—Na1	81.12 (5)
O4^ii^—Na1—O7^iv^	80.02 (5)	Na2—O7—H7B	117.7
O4^iii^—Na1—O7^iv^	99.98 (5)	Na1—O7—H7B	119.5
O2^ii^—Na1—O7^iv^	97.70 (5)	Na2—O7—H7A	120.0
O2^iii^—Na1—O7^iv^	82.30 (5)	Na1—O7—H7A	116.7
O4^ii^—Na1—O7	99.98 (5)	H7B—O7—H7A	102.2
O4^iii^—Na1—O7	80.02 (5)		

Symmetry codes: (i) *x*, *y*+1, *z*; (ii) −*x*+1, −*y*+2, −*z*+1; (iii) *x*, *y*−1, *z*; (iv) −*x*+1, −*y*+1, −*z*+1; (v) −*x*+1, *y*, −*z*+3/2.

### Hydrogen-bond geometry (Å, °)

**Table:**

*D*—H···*A*	*D*—H	H···*A*	*D*···*A*	*D*—H···*A*
O7—H7A···O1	0.79	2.05	2.835 (2)	172
O7—H7B···O1^vi^	0.80	2.03	2.8104 (19)	168

Symmetry codes: (vi) −*x*+1/2, −*y*+3/2, −*z*+1.
